# Metformin as a promising target for DPP4 expression: computational modeling and experimental validation

**DOI:** 10.1007/s12032-023-02140-4

**Published:** 2023-08-25

**Authors:** Amr Ahmed El-Arabey, Haiyan Zhang, Mohnad Abdalla, Samia T. Al-Shouli, Samia S. Alkhalil, Yi Liu

**Affiliations:** 1 Department of Pharmacology and Toxicology, Faculty of Pharmacy, Al-Azhar University, Cairo, 11751 Egypt; 2 Pediatric Research Institute, Children’s Hospital Affiliated to Shandong University, Jinan, 250022 Shandong China; 3 Shandong Provincial Clinical Research Center for Children’s Health and Disease, Jinan, 250022 Shandong China; 4 Immunology Unit, Department of Pathology, College of Medicine, King Saud University, Riyadh, 11461 Saudi Arabia; 5 Department of Clinical Laboratory Sciences, College of Applied Medical Sciences, Shaqra University, Alquwayiyah, Riyadh Saudi Arabia

**Keywords:** Metformin, DPP4, Cancer, TCGA, Tumor microenvironment, Immune cells

## Abstract

Metformin is a regularly prescribed and low-cost generic medication. Metformin has been proposed as a target for Dipeptidyl-peptidase 4 (DPP4) expression in various clinical disorders. We provide insilco investigations on molecular docking and dynamic modeling of metformin and DPP4 potential interactions. Moreover, we conducted bioinformatic studies to highlight the clinical significance of DPP4 expression and mutation in various types of malignancies, as well as the invasion of different immune cells into the tumor microenvironment. We believe the present proposal’s findings have crucial implications for understanding how metformin may confer health advantages by targeting DPP4 expression in malignancies.

**Graphical abstract:**

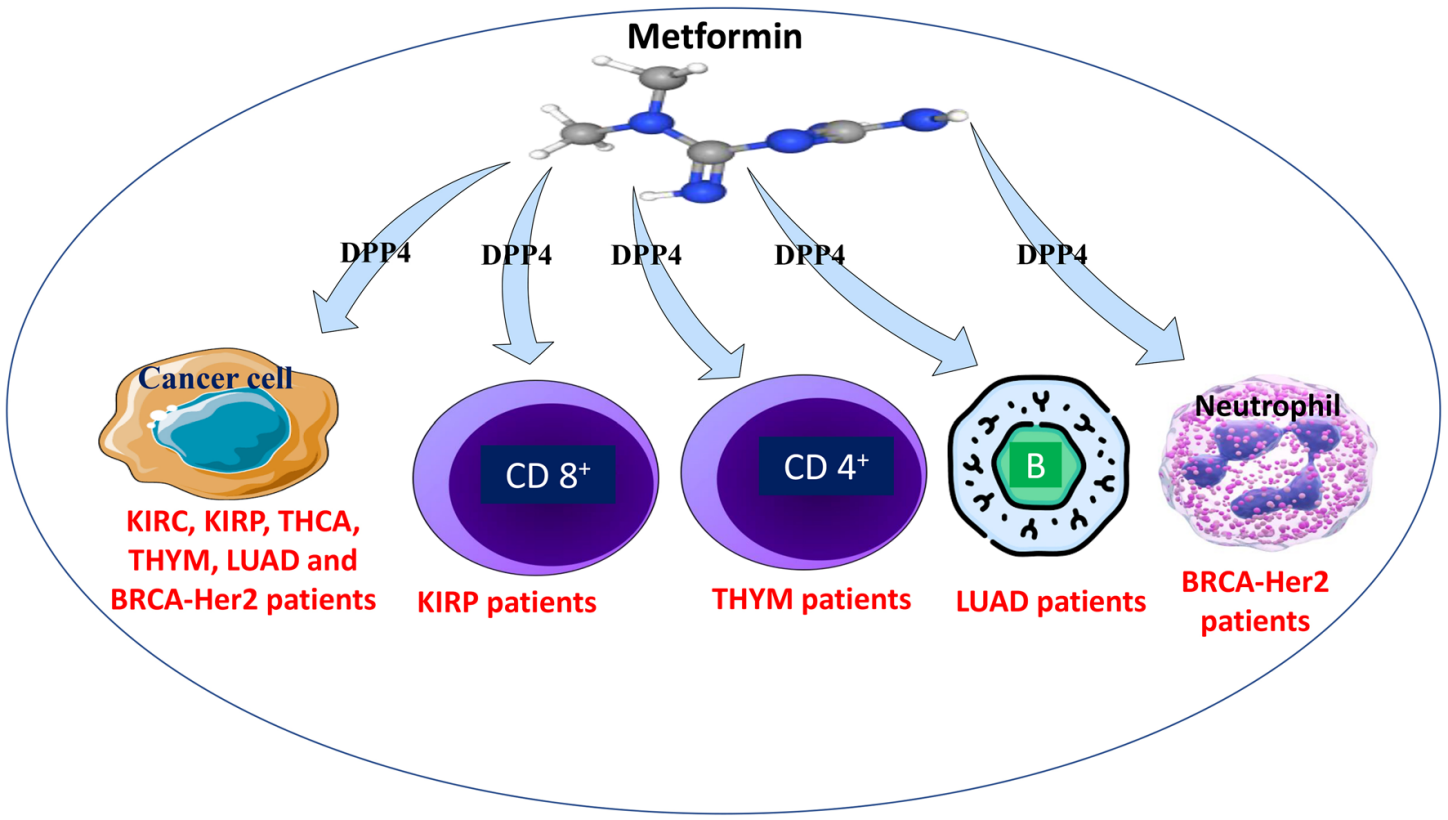

**Supplementary Information:**

The online version contains supplementary material available at 10.1007/s12032-023-02140-4.

## Introduction

Dipeptidyl-peptidase 4 (DPP4) (CD26) is a glycoprotein of 110 kDa found on the surface of the kidney, liver, pancreas, and plasma cells, as well as in the circulation as a soluble form. The DPP4 degrades *N*-terminal dipeptides from a wide range of substrates, such as incretin hormones, growth factors, cytokines, and neuropeptides. DPP4 expression is significantly changed in various clinical situations, including cancer, inflammation, obesity, and diabetes. Furthermore, as a type II transmembrane protein, DPP4 is known to be cleaved from the cell membrane in a cell-type-specific way by several metalloproteases. Thus, DPP4 is important in signaling and immune cell activation, and its dysregulated synthesis and release is linked to a number of illnesses. Since the introduction of DPP4 inhibitors as a promising therapy for type 2 diabetes mellitus patients, the relevance of DPP4 has grown significantly in the scientific and medical communities [1–3]. DPP4 does not influence transcription factors or epigenetics since it is not found in the nucleus of kidney cells. Soluble DPP4, rather than membrane-bound DPP4, may indirectly regulate hemoglobin gene expression in the cytoplasm [4]. DPP4 contains proteolytic action and helps to regulate hemoglobin gene expression. The addition of sitagliptin dramatically boosted DPP4 expression in normal and cancerous kidney cells. These data imply that DPP4 affects hemoglobin gene expression and may play a key role in renal function maintenance [4]. The membrane-bound form of DPP-4 have an essential role in integrin beta-1 and ECM interactions, via which integrins govern cytoskeletal structure and intracellular signaling cascades [5–8]. Interleukin (IL) 12, which is important in developing naive T cells into the Th1 subtype, can also increase DPP4 expression. As a result, DPP4 plays a key role in immune cells stimulation [9]. Furthermore, DPP4, which is produced on CD4^+^ helper/memory T cells and membrane-bound, can send a strong co-stimulatory T cell activation signal. DPP4 also serves as a receptor for the adenosine deaminase, which helps to coordinate immunoregulatory pathways [10]. Normal epithelial cells, fibroblasts, blood vessels, and resident and invading immune cells make up the tumor microenvironment (TME). Signaling chemicals produced by both tumor and normal cells, as well as the extracellular matrix altered by the tumor’s growth. The TME is essential in developing and metastasis several types of cancer. As a result, modern immunotherapy approaches target inhibitory networks inside the TME [11]. The sDPP4 modulates the action of incretin hormones and chemokines by cleaving dipeptides from peptides that have a proline or alanine at the *N*-terminus. There is long-standing evidence that diverse primary tumors and metastases induce sDPP4 to extent degrees. The sDPP4 may have a tumor-promoting or tumor-suppressing function because these effects and interplay with other molecules. In this regard, circulating sDPP4 has also been employed as a cancer biomarker [12]. However, the relevance of DPP4 expression in many malignancies remains uncertain. In this regard, understanding the connection between DPP4 expression and immune cells inside TME will be critical.

Metformin was authorized by the Food and Drug Administration (FDA) in 1995 as an oral hypoglycemic agent. Metformin has become one of the most regularly prescribed diabetic drugs globally, with exciting potential therapeutic applications. According to several reports, metformin is used to treat cancer, aging, polycystic ovarian syndrome, metabolic syndrome, neurological illnesses, cardiovascular diseases, and obesity. In addition, it is utilized for autoimmune diseases via decreasing macrophage cytokine production [13, 14]. Baggio et al. discovered that metformin reduces the quantity of sDPP4 in bone marrow as a significant source for sDPP4 in mice and humans [15]. However, the effect of metformin on DPP4 expression is yet unclear. Therefore, we suggested in the current proposal to examine the clinical consequence of DPP4 expression on patients survival and the infiltration of immune cells within TME of various malignancies. Besides, studying the molecular dynamic modeling and interaction of metformin as promising a druggable target for human DPP4 as well as its experimental influence on DPP4 expression.

## Materials and methods

### Cell viability analysis and morphological changes

Hek293 Cells were seeded in triplicate (5 × 10^3^/well) on 96-well culture plates with 100 μL of culture medium and incubated for 24 h at 37 °C in humidified air containing 5% CO_2_. The cells were treated for 48 h with metformin at 1, 5, or 10 mmol/L concentrations. Each well received 10 μl of CCK-8 solution was incubated for 3 h at 37 ℃. A SpectraMax i3X microplate reader was used to measure the optical density at 450 nm (Molecular Device). The viability was estimated using the formula: percent = (OD of treatment group/OD of the control group) 100 [16]. Morphological alterations in Hek293 cells were examined using an Olympus microscope after 24 and 48 h in control and metformin (1, 5, or 10 mmol/L)-treated cells.

### RNA isolation and real-time quantitative PCR

Total RNA was isolated from Hek293 cells and reverse-transcribed to cDNA using an RT kit (Takara, Japan) following the manufacturer’s instructions. In a real-time thermal cycler, quantitative PCR (qPCR) was done using Quant Inova SYBR Green PCR Kit (Qiagen, USA) on LC480 on Light Cycler 480 instrument (Roche, USA). GAPDH was used as the internal control, and the expression level of DPP4 was normalized to that of GAPDH. The primers for real-time qPCR analysis are as follow: GAPDH forward, 5′- GTGGACCTGACCTGCCGTCT -3′; GAPDH reverse, 5′- GGAGGAGTGGGTGTCGCTGT -3′; DPP4 forward, 5′-AAGATGGAACTGCTTAGTGG-3′; DPP4 reverse, 5′-TAGAGCTTCTATCCCGATGAC-3′. Each experiment was carried out in triplicate and on three different cell samples [16].

### Molecular docking and molecular dynamic simulation analyses

Maestro 12.3’s “Ligand docking” module was used to conduct molecular docking experiments. The “Receptor Grid Generation” module was utilized before docking to build the active binding site on the DPP4 protein. The van der Waals radius scaling factor was set to 1.0 and the partial charge cutoff was set at 0.25. The remaining settings were left at their default values. The “Extra Precision (XP)” model was used to compare metformin molecular docking to the active binding site of DPP4 with sitagliptin as a control. The binding affinity, as well as the molecular interaction behavior were determined [17]. Molecular Dynamic (MD) simulations were performed using the Schrödinger Desmond module. The water-soaked solvated system was created using the Desmond System Builder tool. The TIP3P model was employed as the solvating system in the experiment. The simulation box was orthorhombic, with periodic boundary conditions 10 from the protein’s surface [18]. Counterions were supplied in sufficient quantities to neutralize the water-soaked solvated system. The addition of 0.15 M NaCl to the simulation panel preserved the isosmotic state. Before beginning the simulation, an equilibration procedure was carried out until the system reached a steady condition. The simulation was run for 100 ns at a temperature of 310 K and an ambient pressure of 1.013 bar, respectively. A simulation interaction figure was used to comprehensively investigate the MD simulation data. The root mean square deviation (RMSD) of the DPP4-metformin complex, the DPP4’s root mean square fluctuation (RMSF), the DPP4-ligand interaction figure, the interacting amino acid residues with the ligand in each trajectory frame, and the trajectory of different ligand properties were all investigated in comparison to sitagliptin as the control [17, 19].

### Bioinformatic analyses

#### Analysis of drugs targeting DPP4

TISIDB is an online portal for tumor and the interactions of immune cells that allows researchers to use literature and data analysis to cross-check the importance of a gene of interest in tumor-immune interactions. TISIDB was used as a bioinformatic tool in the current proposal to collect high-throughput data analysis from the drug bank database on medicines targeting DPP4 [20].

#### Analyses of DPP4 expression and mutations in cancers

Tumor-Infiltrating Immune Cells (TIMER) is a comprehensive resource for studying immune infiltrates in different forms of cancer. It assesses the number of immune infiltrates using several immune deconvolution methods and allows for an in-depth investigation into tumor immunological, clinical, and genetic aspects. In the current study, TIMER was used as a bioinformatic tool to build DPP4 mutation module that assesses gene expression changes across mutant statuses. This mutation module presents a heatmap with log2 fold changes in DPP4 expression for each cancer type [21]. In contrast, the differential gene expression module was used to investigate the differential expression of DPP4 between tumor and surrounding normal tissues in all The Cancer Genomic Atlas (TCGA) malignancies. Box plots are used to depict the distributions of DPP4 expression levels. The Wilcoxon test’s statistical significance is shown by the number of stars (*P-value < 0.05; **P-value < 0.01; ***P-value < 0.001). When normal data is available, we may identify DPP4 up- or down-regulation in tumors compared to normal tissues for each cancer type, displayed in gray columns [22].

#### DPP4 outcome analyses in various clinical conditions within cancers

We used TIMER’s gene module to evaluate the clinical importance of tumor DPP4, which allows us to account for multiple variables in a multivariable Cox proportional hazard model. Clinical conditions of DPP4 expression are variables such as age, stage, gender, race, and purity. TIMER computes the cox regression and displays the normalized coefficient of DPP4 for each model across many cancer types for each clinical scenario. This module will provide *z* score, *P* value, as well as adjusted *P*-value [23]. We employed DepMap as a bioinformatic to accelerate precision cancer medicine to analyze metformin sensitivity based on DPP4 expression. Hundreds of cancer cell line models are being profiled by DepMap experts for genomic information and susceptibility to genetic and small chemical alterations. The objective is to establish a landscape of genetic targets for therapeutic development, identify patients who react to these medicines, and better understandancer’s vulnerabilities by triangulating information from this and other large-scale datasets [24].

#### The impact of DPP4 expression on the infiltration of immune cells within tumor microenvironment

Using the gene module, we demonstrated the relationship between DPP4 expression and the infiltration levels of immune cells including CD8^+^ T cell, CD4^+^ T cell, B cell, macrophage, neutrophil, and dendritic cell in cancer types with relevant clinical outcomes. Scatterplots illustrating the purity-corrected partial Spearman’s rho value and statistical significance were constructed and displayed. The gene expression levels against tumor purity are always presented on the left-most panel. DPP4 that are strongly expressed in the microenvironment are predicted to have unfavorable connections with tumor purity, whereas DPP4 that are highly expressed in tumor cells are expected to have the opposite effect [23]. Next, we utilized the survival module to investigate the clinical importance of tumor immune subsets (CD8^+^ T cell, CD4^+^ T cell, macrophage, B cell, neutrophil, and dendritic cell), particularly in cancers with clinical implications for DPP4 expression. In a multivariable Cox proportional hazard model, the survival module has the ability to compensate for many factors. TIMER generates Kaplan–Meier graphs for immune infiltrates to demonstrate survival analysis. This module categorizes levels as low or high and the *P*-value of the log-rank test for comparing the survival curves of two groups is displayed in each Figure [22, 23].

#### Sequence alignment analysis of DPP4

The Molecular Evolutionary Genetics Analysis (MEGA) version has been optimized for use on 64-bit computer platforms for analyzing large datasets. Researchers may study and assess tens of thousands of sequences using Mega. The new MEAG7 version has an improved wizard for constructing time trees as well as new features for forecasting gene duplication events in gene family trees. MEAG7 software was used to create an alignment of DPP4 protein sequences from humans, pigs, bovines, cats, mice, and rats. The conserved amino acids are highlighted in red background, and the arrow represents beta-sheets, while the helix indicates alpha-sheets [25].

## Results

### Metformin inhibits Hek293 cell growth while increasing DPP4 expression

**Fig. 1:**
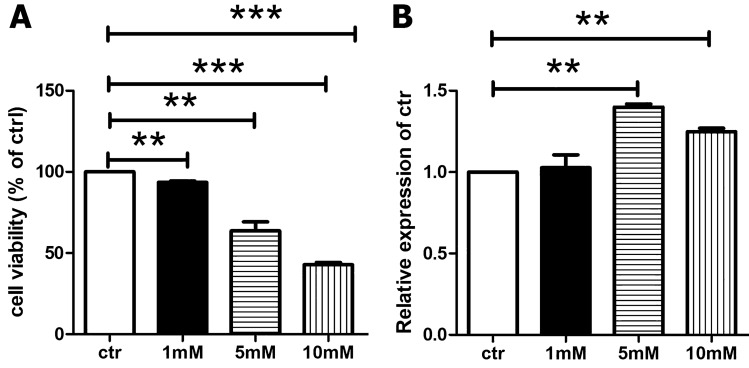
**A**: Cell viability examination of Hek293 cells after 48 h of metformin treatment at 1, 5, or 10 mmol/L doses. **B**: DPP4 expression PCR analysis revealed that metformin enhances DPP4 expression in Hek293 cells

Our results outlined that metformin treatment (1, 5, or 10 mmol/L) significantly decreases cell growth of Hek293 Cells after 48 h period (*P*-value < 0.05) (Fig. 1A). This is not surprising given that reduced proliferation of Hek293 cells was seen after 24 and 48 h with no characteristic morphological alterations (Supplementary Fig. 1). TISIDB analysis was used to identify well-known compounds that target DDP4. We did not find metformin’s Drug Bank accession number (DB00331) among the putative DPP4 targets (Supplementary Fig. 2). Next, we utilized MEAG7 software to study the alignment of DPP4 sequences from humans, pigs, bovines, cats, mice, and rats (Supplementary Figs. 3 and 4). We next use PCR to examine the effect of metformin on DPP4 expression in Hek293 cells. Our findings show that metformin promotes DPP4 expression in Hek293 cells (Fig. 1B).

### Molecular docking and molecular dynamics simulation analyses of metformin with DPP4

**Fig. 2:**
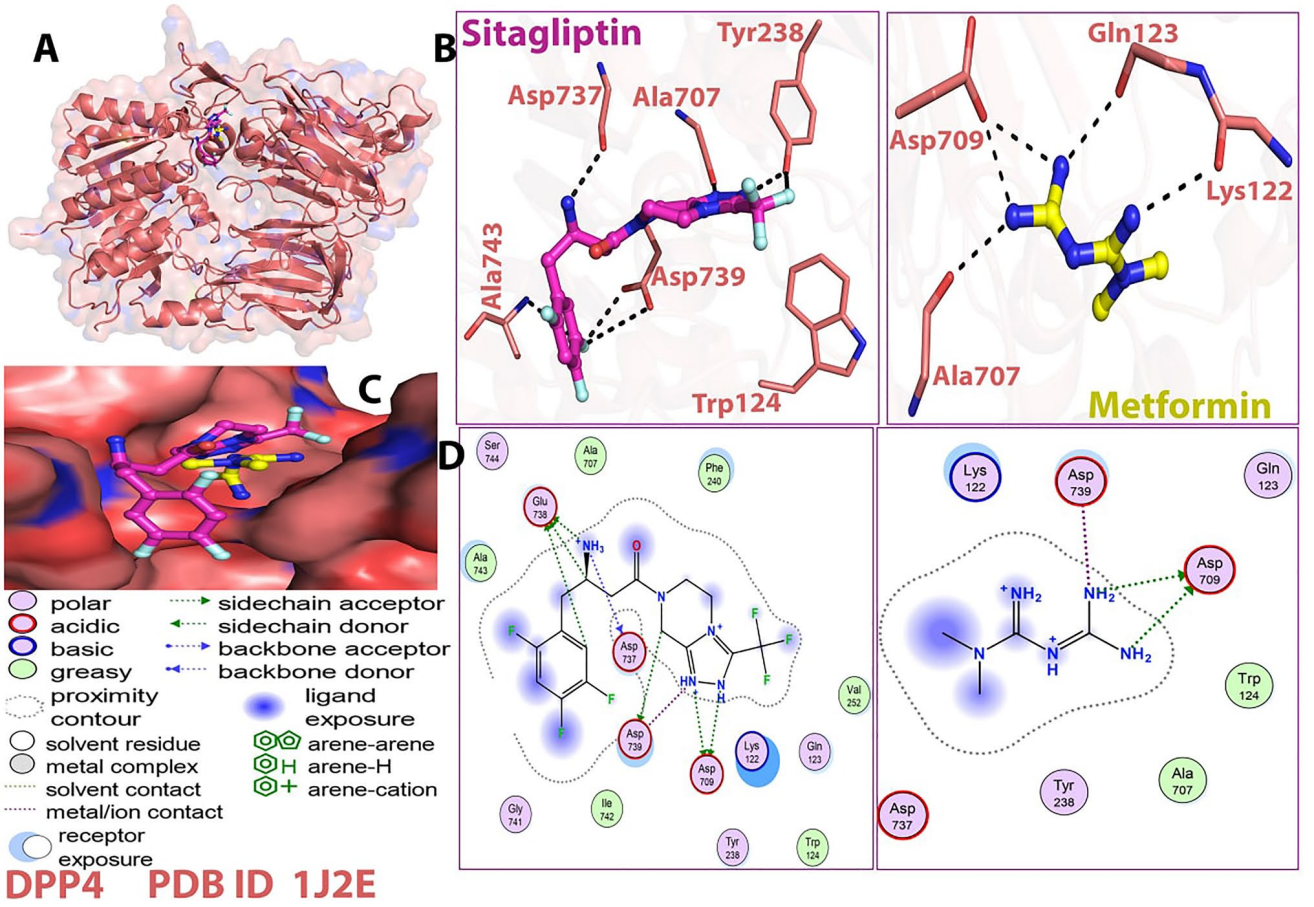
Visualization of 3D ligand interactions of metformin and sitagliptin with DPP4

We used molecular docking and MD modeling to confirm that metformin is a direct target of DPP4 expression. Figure 2 depicts metformin’s two-dimensional and three-dimensional ligand interactions with DPP4 compared to sitagliptin as a control. When metformin was compared to sitagliptin as a control, the QSAR analysis revealed some critical elements connected to its chemical structure. Table 1 shows the QSAR ratings for metformin and sitagliptin. Metformin has a smaller surface area, volume, Log *P*, refractivity, polarizability, mass, dipole moment, and total energy than sitagliptin. Further, metformin has greater hydration energy than sitagliptin. Tables 2 and 3 summarize the interactions produced between distinct atoms of metformin and sitagliptin and the DPP4, as well as the interaction type, interacting lengths, and interacting energy (kcal/mol). Similarly, to sitagliptin, metformin interacts with DPP4 amino acid residues such as OD2-ASP-709 and OD1-ASP-739 through H donor and ionic interactions.

**Table 1:** QSAR rating for optimized compounds

Function	Metformin	Sitagliptin
Surface area (Approx) (Å^2^)	173.14	488.21
Surface area (Grid) (Å^2^)	312.10	587.29
Volume (Å^3^)	455.30	984.93
Hydration energy (Kcal/mole)	− 19.24	− 6.61
Log *P*	− 1.25	5.12
Refractivity (Å^3^)	18.86	49.33
Polarizability (Å^3^)	10.23	32.70
Mass (amu)	125.13	407.32
Total energy (kcal/mol)	8.15114	15.5738
Dipole moment (Debye)	0.3668	2.446
RMS gradient (kcal/Å mol)	0.0949	0.09881

The QSAR analysis showed certain crucial aspects related to the chemical structures of Metformin when compared to sitagliptin as a control. Table 2 shows the QSAR ratings for metformin and sitagliptin. Surface area, volume, hydration energy, log *P*, refractivity, polarizability, mass, total energy, dipole moment, and RMS gradient are all investigated. Metformin has a smaller surface area, volume, Log *P*, refractivity, polarizability, mass, dipole moment, and total energy than sitagliptin. Furthermore, metformin has a greater hydration energy than sitagliptin

**Table 2:** Details of interactions type between the metformin and the amino acid residues of DPP4:1j2e/metformin

Ligand	Receptor	Interaction	Distance	E (kcal/mol)
N4 6	OD2 ASP 709 (A)	H-donor	2.93	− 7.5
N5 7	OD2 ASP 709 (A)	H-donor	3.17	− 3.3
N4 6	OD2 ASP 709 (A)	ionic	2.93	− 4.9
N5 7	OD2 ASP 709 (A)	ionic	3.17	− 3.5
N5 7	OD1 ASP 739 (A)	ionic	3.23	− 3.1

**Table 3:** Details of interactions type between the sitagliptin and the amino acid residues of DPP4: 1j2e/sitagliptin

Ligand	Receptor	Interaction	Distance	E (kcal/mol)
N3 4	OD1 ASP 709 (A)	H-donor	2.62	− 12.3
N5 6	OD2 ASP 709 (A)	H-donor	2.69	− 20.6
C3 14	OD1 ASP 739 (A)	H-donor	3.03	− 1.7
C6 23	OE1 GLU 738 (A)	H-donor	3.00	− 1.2
N4 25	O ASP 737 (A)	H-donor	2.78	− 5.5
N4 25	OE2 GLU 738 (A)	H-donor	2.70	− 8.1
C13 41	OE1 GLU 738 (A)	H-donor	3.36	− 1.2
N3 4	OD1 ASP 709 (A)	ionic	2.62	− 7.6
N3 4	OD2 ASP 709 (A)	ionic	3.32	− 2.7
N3 4	OD1 ASP 739 (A)	ionic	3.21	− 3.2
N5 6	OD1 ASP 709 (A)	ionic	2.95	− 4.8
N5 6	OD2 ASP 709 (A)	ionic	2.69	− 6.9
N4 25	OE1 GLU 738 (A)	ionic	3.30	− 2.8
N4 25	OE2 GLU 738 (A)	ionic	2.70	− 6.8

**Fig. 3:**
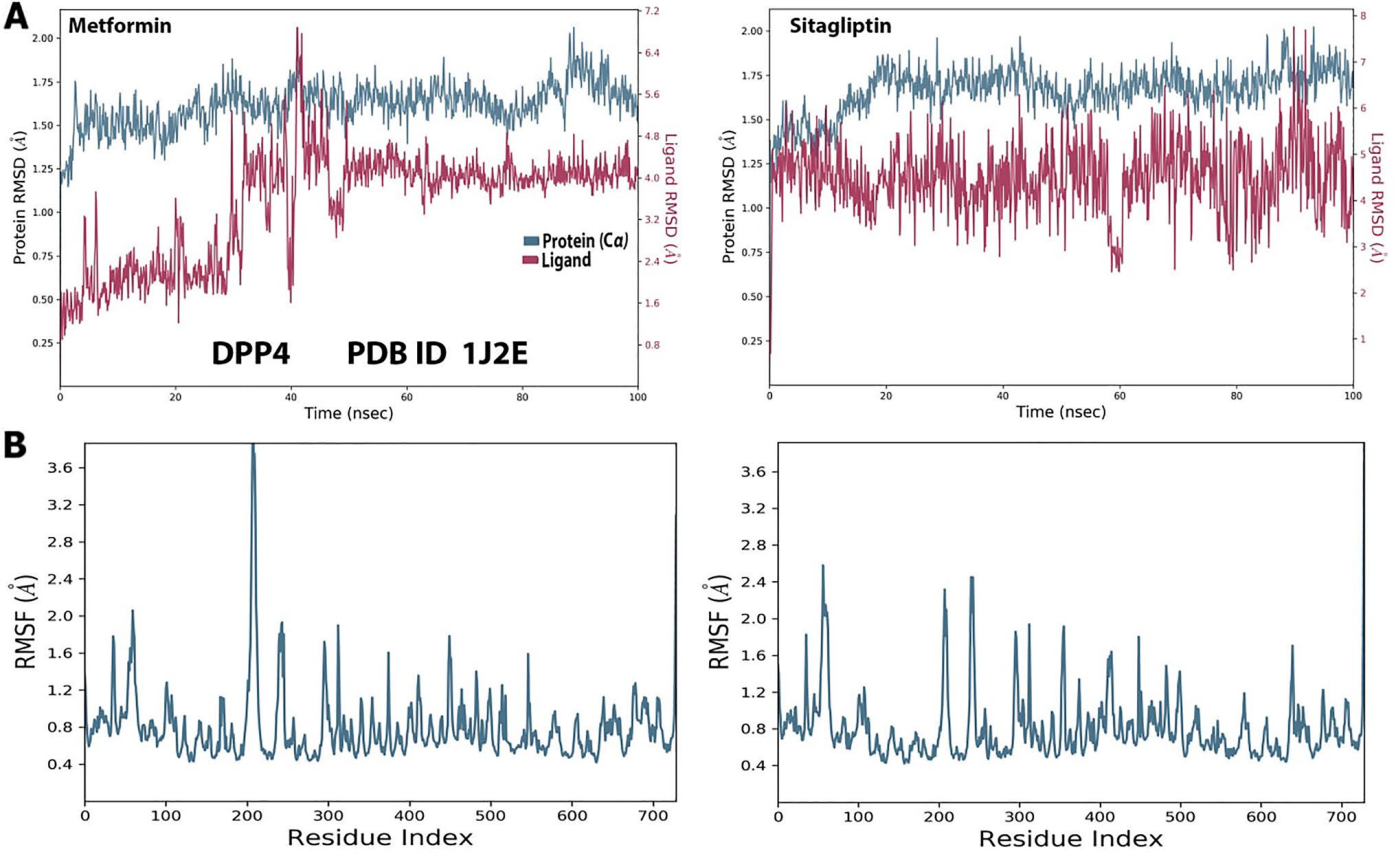
**A**: RMSD of protein–ligand interaction during 100 ns MD simulation for metformin and sitagliptin with DPP4. **B**: Metformin and sitagliptin RMSF of protein–ligand interaction during 100 ns MD simulation with DPP4

To compare metformin and sitagliptin with DPP4, MD simulations were run for 100 ns. Following the MD simulations, the RMSD was calculated. RMSD may be used to investigate changes in individual atom states using their starting state as a reference. This implies that the ligands’ initial docked posture at the target protein’s binding site will be utilized as the reference frame, and the mobility of this original frame during the MD simulation will be analyzed in terms of time. For the DPP4, the RMSD values are given on Y-axis (left side) in (Fig. 3A). Throughout the simulation, metformin’s interaction with DPP4 is similar to sitagliptin’s interaction with DPP4, with both having an RMSD of roughly 2.0 (Fig. 3A). The RMSF data reveals information about constrained shifts in protein chains. The RMSF plot shows the portions of the protein that differ the greatest from the reference. Figure 5 depicts the graphical RMSF data for the DPP4 with metformin and sitagliptin obtained by the MD simulation. Visual investigation indicated that the protein terminals move more than other locations in all protein–ligand complexes. The RMSF value of metformin should not change significantly from the RMSF value of sitagliptin (Fig. 3B).

**Fig. 4:**
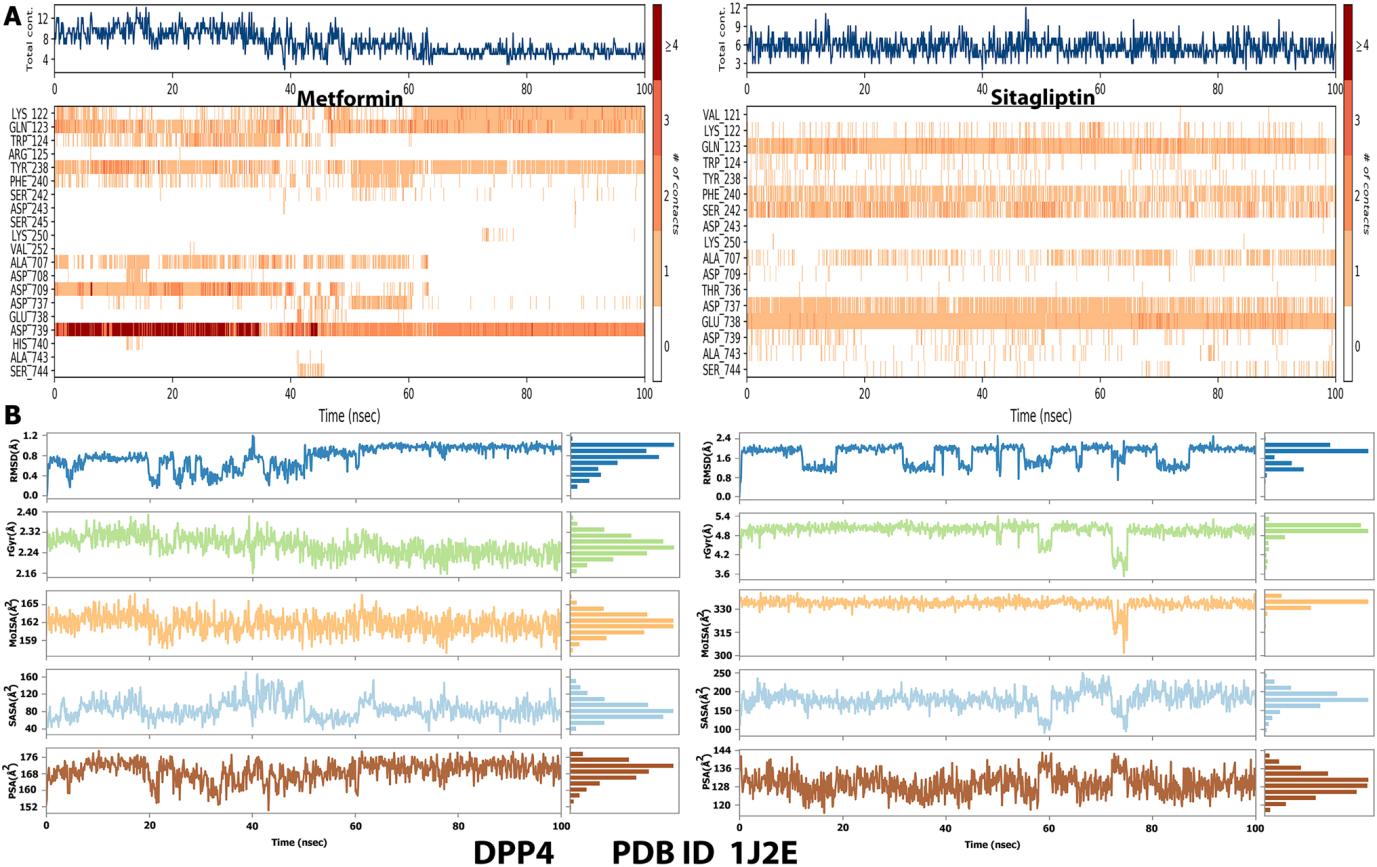
**A**: The trajectory of different properties of metformin and sitagliptin with DPP4 during the 100 ns of MD simulations. **B**: Timeline interaction data of the amino acids of the DPP4 with metformin and sitagliptinthroughout the entire 100 ns MD simulations

**Fig. 5:**
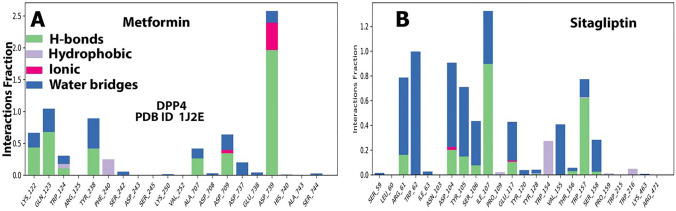
Illustration of the interaction of the crucial amino acids of the DPP4 protein with Metformin (**A**) and sitagliptin (**B**) throughout the 100 ns MD simulations

The protein–ligand interaction plot and schematic depiction **(**Figs. 4 and 5**)** verify the MD simulation study’s findings, as the amino acids residues of DPP4 were discovered to interact with metformin and sitagliptin during docking during the 100 ns MD simulations. In fact, our data revealed that the amino acids residues of DPP4 identified to interact with sitagliptin are more significant than the amino acids residues of DPP4 discovered to interact with metformin. Nonetheless, metformin and sitagliptin have similar interactions with ASP-709 and ASP-739 to varying degrees (Fig. 4A). Next, (Fig. 4B) illustrates the graphical findings for metformin and sitagliptin with DPP4. The radius of gyration (rGyr) reveals the compactness of a structure, with large variations indicating less stability. The molecular surface area (MolSA) is a geometric surface attribute in which the value of MolSA and van der Walls surface area are equal. The solvent accessible surface area (SASA) (Fig. 4B) displays the characteristics of metformin in contrast to sitagliptin. The RMSD of metformin is between 0.5 and 1.1, while the equilibrium is between 0.9 and 1.1 Å. The RMSD of sitagliptin is between 0.6 and 2.2 Å, while the equilibrium is between 0.9 and 1.6 Å (Fig. 4B). The Fig. 5 highlights the interactions of metformin and sitagliptin with the DPP4 complex. Figure 5A validates metformin’s MD simulation results, showing that it interacted with LYS122, GLN123, TRP124, TYR238, PHE240, SER242, ALA707, ASP709, ASP737, GLU738, and ASP739. Figure 5B validates the MD simulation findings of sitagliptin, showing that it interacted with ARG61, TRP62, ILE63, ASP104, TYR105, SER106, ILE107, GLU117, TYR120, TYR128, TYR154, VAL155, THR156, TRP157, SER158, and TRP216 (Supplementary Fig. 3).

### DPP4 has clinical significance in a variety of cancers

**Fig. 6:**
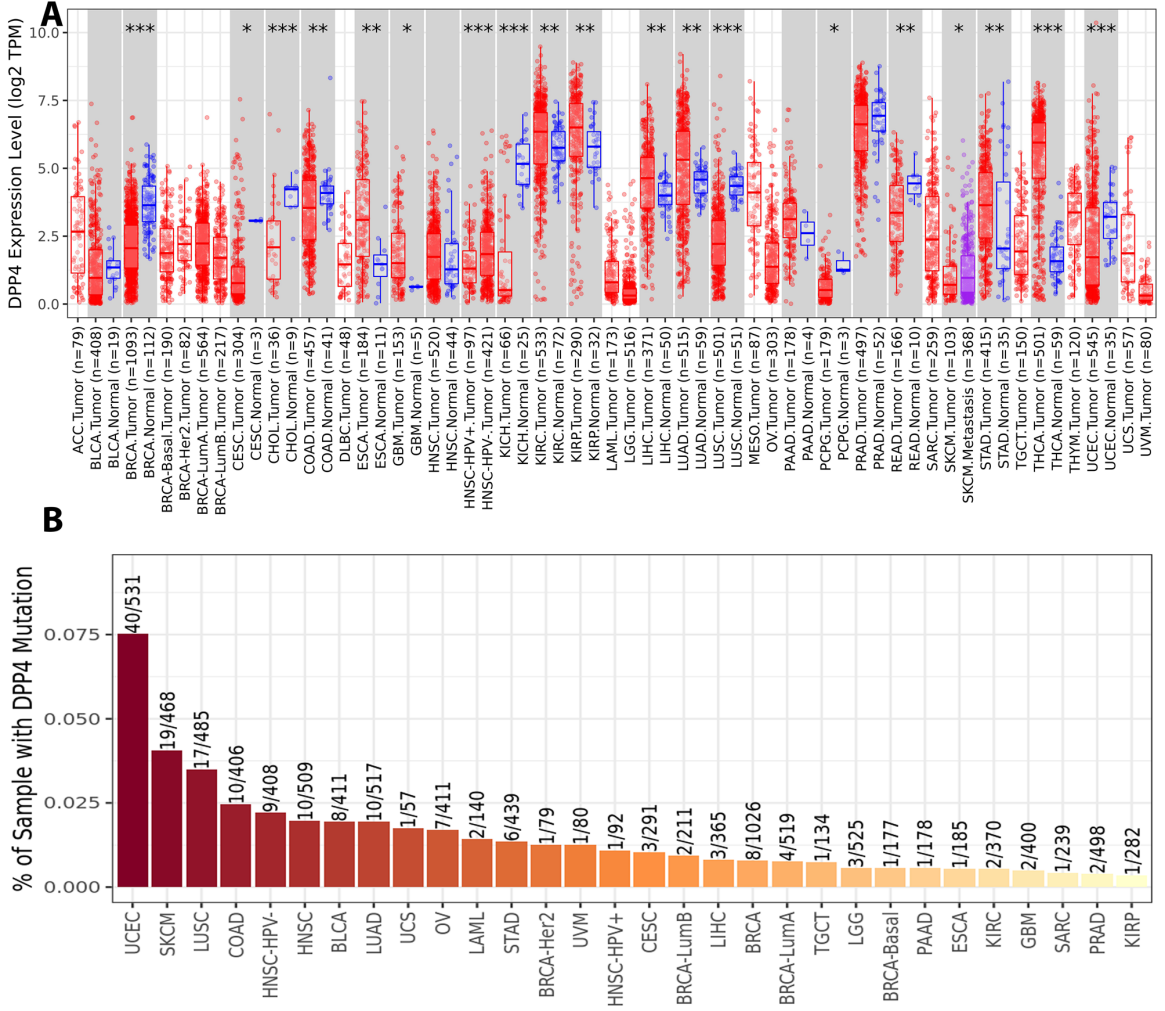
**A**: Bioinformatic analysis of DPP4 differential expression in different sort of cancer of TCGA data via TIMER. **B**: The DPP4 mutation module analysis of TCGA data via TIMER. The DPP4 was most mutated in UCEC, SKCM, and LUSC and least mutated in KIRP, PRAD, and SARC

Our bioinformatic analyses of the TCGA using TIMER revealed that the differential expression of DPP4 is significant in 19 distinct types of cancer, including BRCA, CHOL, HNSC-HPV, KICH, LUSC, THCA (*P*-value < 0.001), COAD, ESCA, KIRC, KIRP, LIHC, LUAD, READ, STAD (*P*-value < 0.01), CESC, GBM, PCPG, SKCM, and UCEC (*P*-value < 0.05) (Fig. 6A). The DPP4 mutation module analysis demonstrated that DPP4 was most mutated in UCEC, SKCM, and LUSC and least mutated in KIRP, PRAD, and SARC (Fig. 6B). Following that, we used TIMER to determine clinical consequences of DPP4 expression depending on purity, gender, age, stage, and race in TCGA data from various types of cancer. First, in terms of purity and gender, the findings showed that DPP4 has a clinically significant influence in KIRC, KIRP, THCA, and THYM (*P*-value < 0.05) (Supplementary files 5 and 6). Second, the data in terms of age revealed that DPP4 has a clinically significant impact in KIRC, KIRP, and LUAD (*P*-value < 0.05) (Supplementary file 7). Third, the findings showed that DPP4 has a clinically significant influence on KIRC, BRCA-Her2, LUAD, THCA, and THYM (*P*-value < 0.05) in term of stage (Supplementary file 8). Finally, the TCGA data showed that DPP4 had a clinically significant effect on KIRC, KIRP, LUAD, THCA, and THYM in the context of race (*P*-value < 0.05) (Supplementary file 9). Overall, our results showed that DPP4 had a clinically significant influence in all of the analyzed parameters purity, gender, age, stage, and race in KIRC TCGA data (Supplementary files 5–9). This is why it is not unexpected to notice the encouraging findings about metformin sensitivity to distinct kidney cancer cell lines with the association of DPP4 expression by utilizing DepMap (Supplementary Fig. 10).

**Fig. 7:**
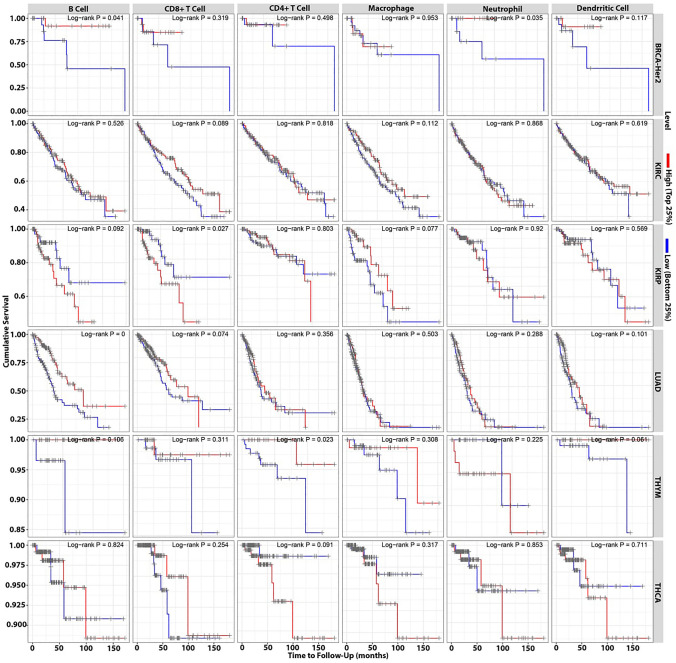
The survival analysis of TCGA data using TIMER reveals the clinical significance of tumor immune subsets on patient survival, such as neutrophils in BRCA-Her2, B cells in LUAD, CD8^+^ T cells in KIRP, and CD4^+^ T cells in THYM

**Fig. 8:**
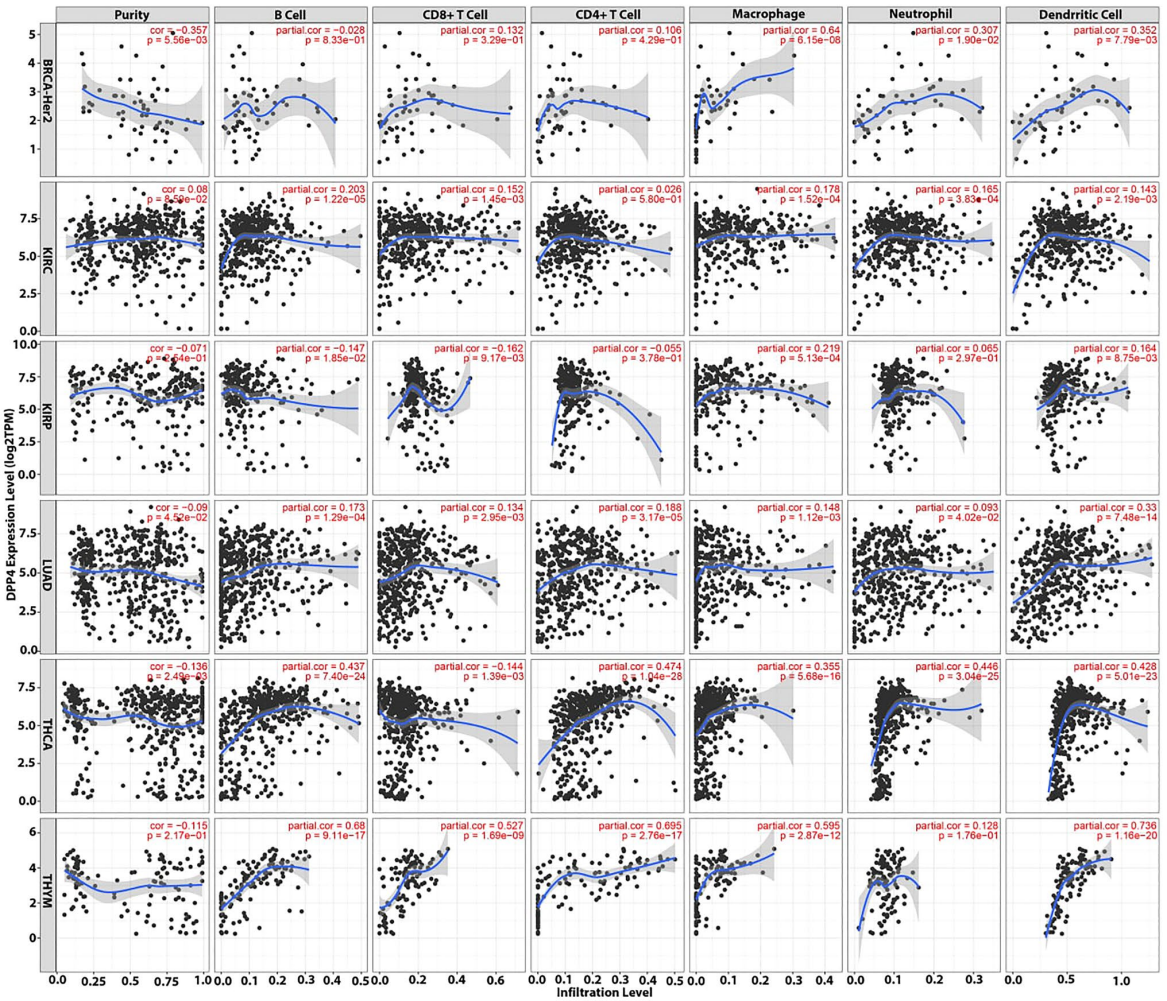
Bioinformatic analysis of the association between DPP4 expression and immune cell infiltration revealed that DPP4 expression is positively linked with neutrophil infiltration in BRCA-Her2 patients, B cell infiltration in LUAD patients, and CD4^+^ T cell infiltration in THYM patients. In contrast, DPP4 expression is negatively associated to CD8^+^ T cells in KIRP patients

The survival module was used to look at the clinical significance of tumor immune subsets (CD8^+^ T cell, CD4^+^ T cell, macrophage, B cell, neutrophil, and dendritic cell) in BRCA-Her2, KIRC, KIRP, LUAD, THCA, and THYM. Our findings show that neutrophils in BRCA-Her2, B cells in LUAD, CD8^+^ T cells in KIRP, and CD4^+^ T cells in THYM have a clinical influence on patient survival (Fig. 7). Besides, our data highlighted that DPP4 is a potential target for immune cells such as neutrophil, B cell, CD8^+^ T cell, and CD4^+^ T cell. In this sense, DPP4 expression is positively associated with neutrophil infiltration in BRCA-Her2 patients, B cell infiltration in LUAD patients, and CD4^+^ T cell infiltration in THYM patients. In contrast, in KIRP patients, DPP4 expression is inversely related to CD8^+^ T cells (Fig. 8).

## Discussion

The DPP4 regulates energy metabolism, inflammation, and immunological function and hence plays a role in a variety of physiological and pathological processes [26]. Metformin is a biguanide that has become one of the most extensively used drugs. This component’s uses include but are not limited to, lowering blood glucose, weight reduction, and polycystic ovarian syndrome. Additional studies on probable indications have emerged, showing that this drug can be used for other purposes. Metformin has anti-inflammatory actions via many pathways, making it a viable therapeutic target for inflammatory illnesses. Furthermore, because inflammation is a major component of tumors incidence and progression, targeted inflammatory management can help with both cancer prevention and therapy [27]. The interaction between metformin and DPP4 as well as the effect of metformin on DPP4 expression, remain unclear. Further, the clinical significance of DPP4 expression in various types of malignancies, as well as its involvement in immune cells infiltration inside TME, remain unanswered concerns.

Metformin therapy dramatically reduces cell proliferation in Hek293 cells, as indicated in this study. These findings corroborate previous findings that metformin regulates cell size, proliferation, and protein synthesis by downregulating the expression of AMP-activated protein kinase (AMPK) and Cyclin-Dependent Kinase Inhibitor 1A (p21) upstream of the mammalian target of rapamycin (mTOR) pathway [28]. Metformin was recently approved to target the essential source of sDPP4 in mice and humans [15]. Our findings show that metformin is a potential target of DPP4 expression for the first time because we did not identify metformin among the putative DPP4 targets. We predicted the hypothesis using molecular docking and MD modeling using sitagliptin as a control, and we tested it experimentally using PCR analysis. Substantially, the current investigation demonstrated that metformin increases DPP4 expression in the HEK293 cell line. Similarly, a prior study found that sitagliptin dramatically enhanced the expression of DPP4 in HEK293 cells, which regulates the expression of hemoglobin genes and plays an important role in renal function maintenance [4]. This explains why metformin interacts with DPP4 amino acid residues like OD2-ASP-709 and OD1-ASP-739 through H donor and ionic interactions like sitagliptin. Besides, metformin’s RMSF value should not differ considerably from sitagliptin’s RMSF value. The RMSF measures a specific atom’s or group of atoms’ displacements relative to the reference structure, averaged across the number of atoms [29]. The RMSF value is critical for determining the fluctuations of the side chains of residues during MS simulations [30].

The DPP-4’s enzymatic activity has been identified as a crucial mediator for chemokines, incretins, and neuropeptides [31, 32]. DPP4 also has an effect on different lung problems such asthma, chronic obstructive pulmonary disease, pulmonary fibrosis and middle east respiratory syndrome [26]. However, more study is required to properly investigate the clinical significance of DPP4 expression in a wide range of malignancies as well as in the TME. The analysis of TCGA data is well recognized as the main modern technique for present and future research to examine the complicated relationships within TME [33]. Our bioinformatic analyses of the TCGA data indicated that DPP4 expression differs in a variety of malignancies. Nonetheless, the data revealed that DPP4 had the most significant influence on KIRC in many variables such as purity, gender, age, stage, and race. These findings were consistent with a recent randomized trial that found metformin paired with intensive-exercise diet therapy significantly improves glucose and lipid metabolism in renal cancer patients with diabetes and effectively improves 12-month progression-free survival. However, further experiments are needed before clinical applicability [34]. In KIRP, THCA, and THYM patients, the DPP4 had the second highest influence order. In clinical trials, metformin was used to treat KIRP and KIRC patients ‘’NCT02495103’’ and THCA patients ‘’NCT01341886, NCT04298684, NCT03109847, NCT05468554, NCT03183752’’ (https://clinicaltrials.gov/). DPP4 may be a target for immunological cells such as neutrophils in BRCA-Her2 patients, B cells in LUAD patients, and CD8^+^  T cells in KIRP patients. Numerous studies have been conducted to investigate the effect of metformin on BRCA-Her2, LUAD patients. These studies reveal that metformin suppresses cancer development and metastasis in these patients [35, 36]. In this sense, several ongoing and completed clinical trials highlighting the impact of metformin for BRCA-Her2 patients ‘’NCT03238495, NCT02488564, NCT04899349, NCT01477060, NCT01101438, NCT01042379’’ and for LUAD patients ‘’NCT01997775, NCT02823691, NCT04931017, NCT02115464, NCT02145559, NCT04001725, NCT01578551, NCT02431676’’(https://clinicaltrials.gov/). Further, DPP4 expression is related with CD4^+^ T cell infiltration in THYM patients. Hence, multiple research and ongoing clinical trials have shown that metformin may enhance the overall survival of THYM patients [37]. Consequently, metformin is a potential target for DPP4 expression in many malignancies and can modify immune cells infiltration into TME. In addition, our bioinformatics findings reveal a new road for metformin’s therapeutic application adventure against cancer.

### Supplementary Information

Below is the link to the electronic supplementary material.

Supplementary file1 (JPG 109 KB)

Supplementary file2 (JPG 115 KB)

Supplementary file3 (JPG 2290 KB)

Supplementary file5 (JPG 3504 KB)

Supplementary file5 (XLSM 12 KB)

Supplementary file6 (XLSM 12 KB)

Supplementary file7 (XLSM 12 KB)

Supplementary file8 (XLSM 12 KB)

Supplementary file9 (XLSM 13 KB)

Supplementary file10 (JPG 160 KB)

Supplementary file11 (DOCX 18 KB)

## Abbreviations

ACC: Adrenocortical carcinoma
BLCA: Bladder urothelial carcinoma
BRCA: Breast invasive carcinoma
CHOL: Cholangiocarcinoma
COAD: Colon adenocarcinoma
DLBC: Diffuse large B-cell lymphoma
DPP4: Dipeptidyl-peptidase 4
ESCA: Esophageal carcinoma
FDA: Food and drug administration
GBM: Glioblastoma multiform
HNSC: Head and neck cancer
KICH: Kidney chromophobe
LGG: Lower grade glioma
LIHC: Liver hepatocellular carcinoma
LUAD: Lung adenocarcinoma
LUSC: Lung squamous cell carcinoma
MEGA: Molecular evolutionary genetics analysis
OV: Ovarian serous cystadenocarcinoma
PAAD: Pancreatic adenocarcinoma
READ: Rectum adenocarcinoma
PRAD: Prostate carcinoma
RMSD: The root mean square deviation
RMSF: Root mean square fluctuation
SARC: Sarcoma
SCNA: Somatic copy-number alterations
SKCM: Skin cutaneous carcinoma
STAD: Stomach adenocarcinoma
TCGA: The cancer genomic atlas
THYM: Thymoma
TIMER: Tumor IMmune estimation resource
TME: Tumor microenvironment
UCEC: Uterine corpus endometrial carcinoma
UCS: Uterine carcinosarcoma
