# Supplementary figures and images for: Metformin as a promising target for DPP4 expression: computational modeling and experimental validation
Source: Med Oncol. 2023 Aug 25;40(10):277. doi: 10.1007/s12032-023-02140-4 (PMC10457412; doi:10.1007/s12032-023-02140-4)

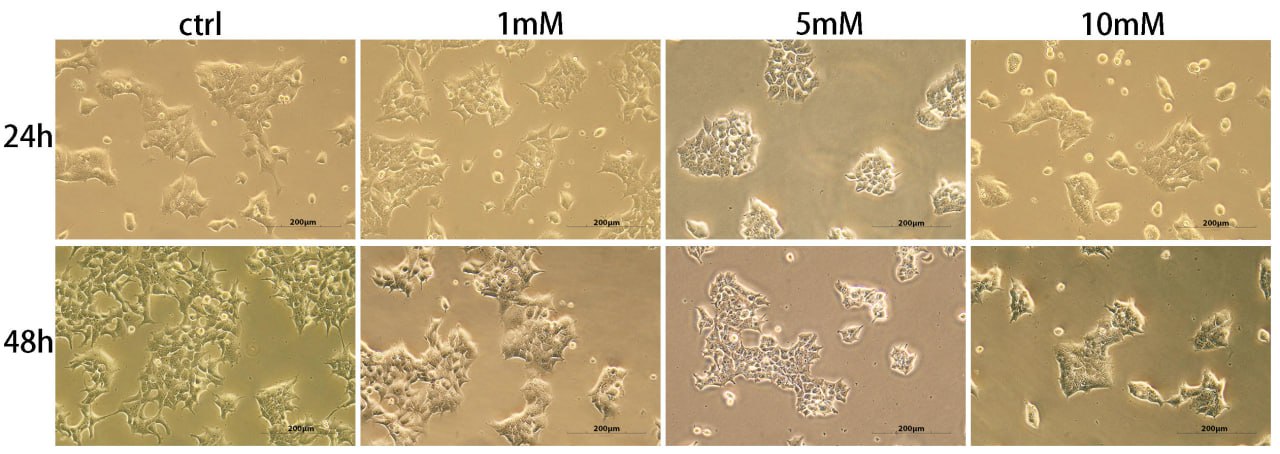

Supplement: Supplementary file 1 — Supplementary file1 (JPG 109 KB) [file 12032_2023_2140_MOESM1_ESM.jpg]

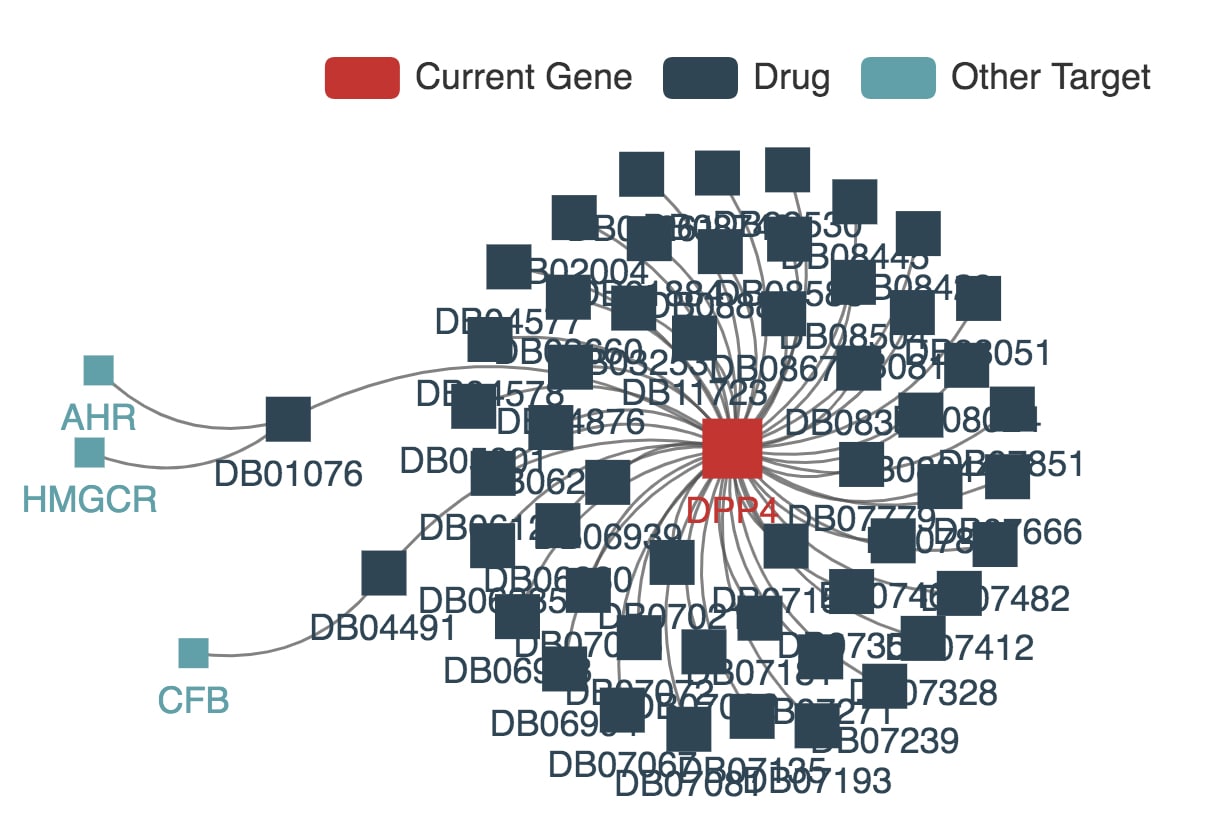

Supplement: Supplementary file 2 — Supplementary file2 (JPG 115 KB) [file 12032_2023_2140_MOESM2_ESM.jpg]

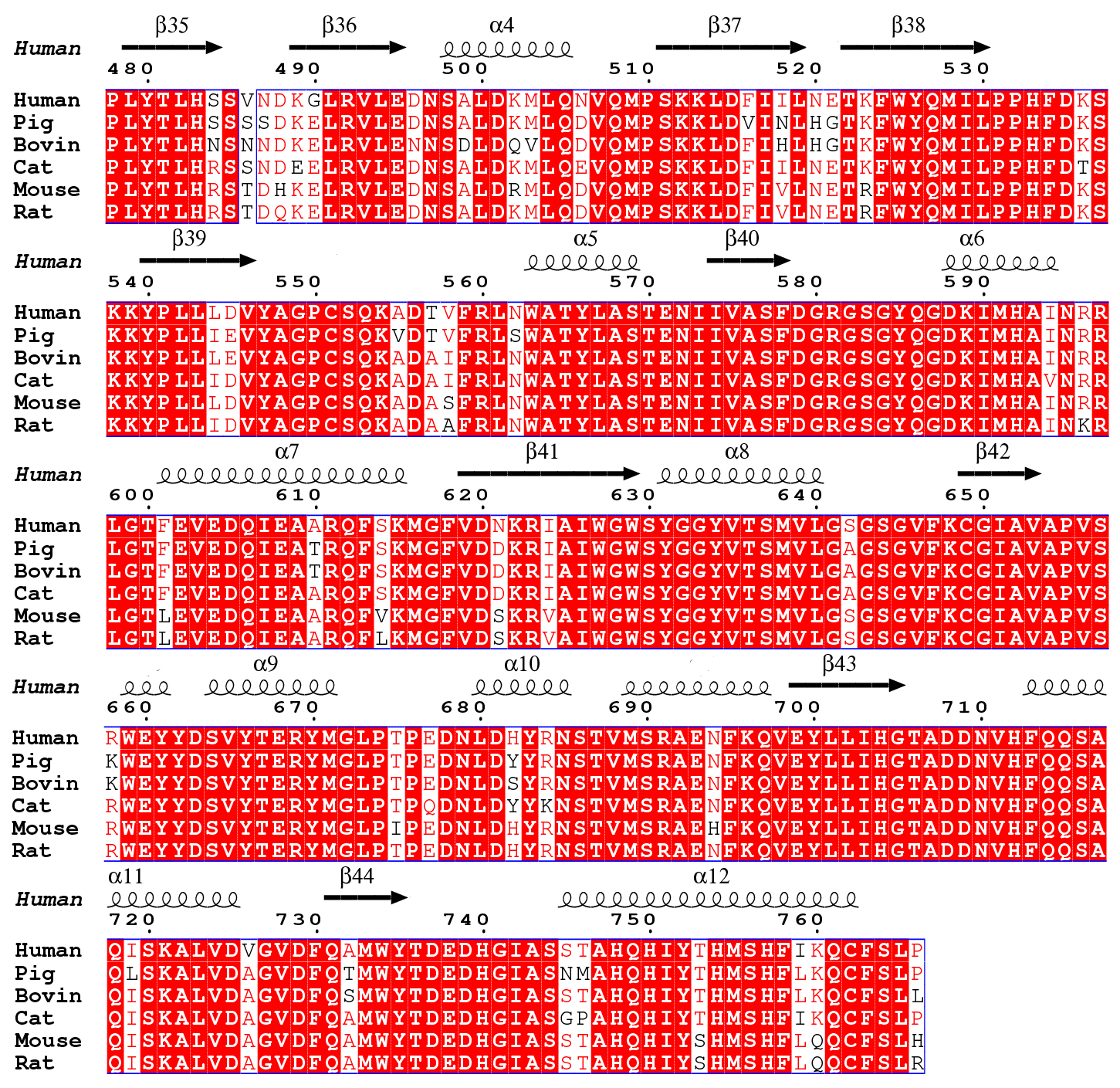

Supplement: Supplementary file 3 — Supplementary file3 (JPG 2290 KB) [file 12032_2023_2140_MOESM3_ESM.jpg]

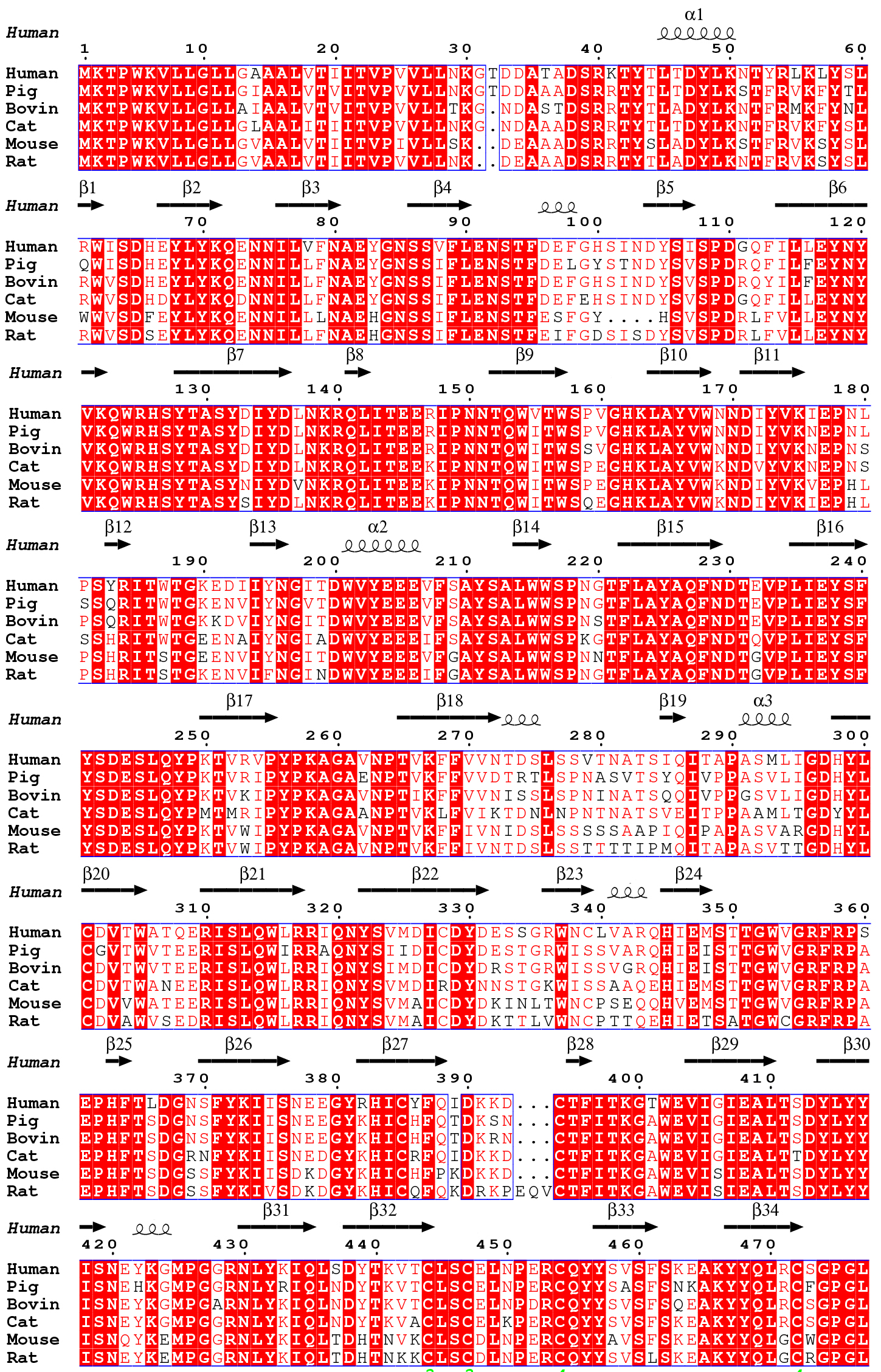

Supplement: Supplementary file 4 — Supplementary file5 (JPG 3504 KB) [file 12032_2023_2140_MOESM4_ESM.jpg]

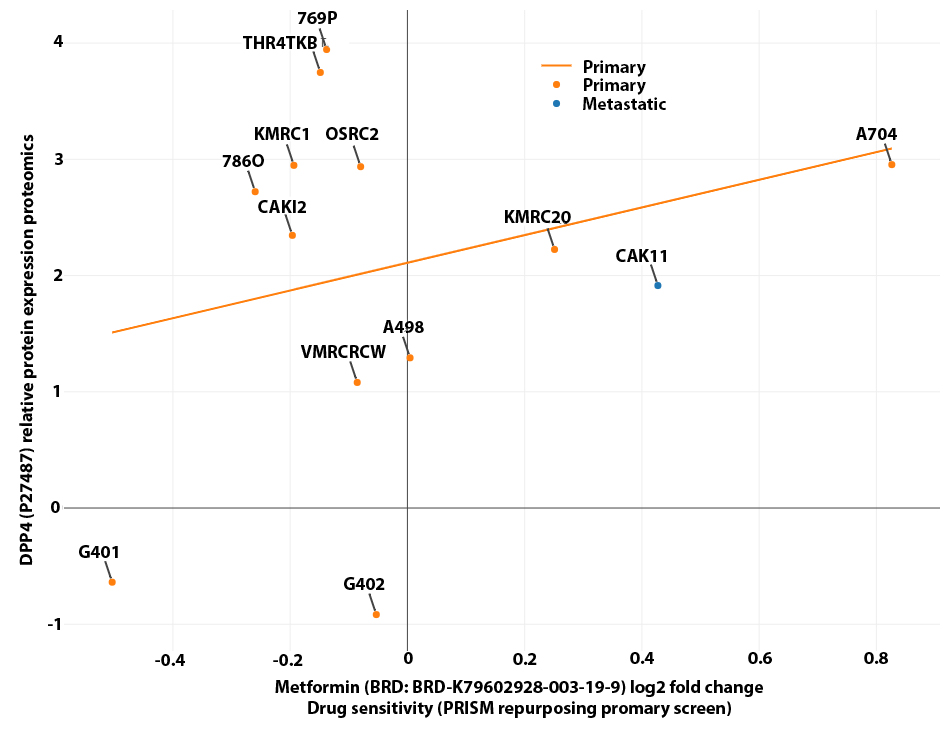

Supplement: Supplementary file 10 — Supplementary file10 (JPG 160 KB) [file 12032_2023_2140_MOESM10_ESM.jpg]
