# Supplementary material for: Metformin as a promising target for DPP4 expression: computational modeling and experimental validation
Source: Med Oncol. 2023 Aug 25;40(10):277. doi: 10.1007/s12032-023-02140-4 (PMC10457412; doi:10.1007/s12032-023-02140-4)
Supplement: Supplementary file 11 — Supplementary file11 (DOCX 18 KB) [file 12032_2023_2140_MOESM11_ESM.docx]

**Supplementary Figure 1:** Morphological characterizations of Hek293 cells after 24 and 48 hours of metformin treatment.

**Supplementary Figure 2:** TISIDB examined the potential DPP4 targets and discovered that metformin's Drug Bank entry number (DB00331) was not among them.

**Supplementary Figure 3 and 4:** Sequence alignment analysis of DPP4 via The Molecular Evolutionary Genetics Analysis (MEGA) version

**Supplementary File 5-9:** Bioinformatics investigation using TIMER to assess clinical effects of DPP4 expression in TCGA data from various kinds of cancer based on purity, gender, age, stage, and race.

**Supplementary Figure 10:** Metformin sensitivity in several kidney cancer cell lines was linked to DPP4 expression using DepMap.
